# Synergistic Myoelectrical Activities of Forearm Muscles Improving Robust Recognition of Multi-Fingered Gestures

**DOI:** 10.3390/s19030610

**Published:** 2019-02-01

**Authors:** Xiuying Luo, Xiaoying Wu, Lin Chen, Yun Zhao, Li Zhang, Guanglin Li, Wensheng Hou

**Affiliations:** 1 Key Laboratory of Biorheological Science and Technology, Ministry of Education, Bioengineering College, Chongqing University, Chongqing 400044, China; 20161902039@cqu.edu.cn (X.L.); x.y.wu@cqu.edu.cn (X.W.); 20161901009@cqu.edu.cn (Y.Z.); lilyzhang@cqu.edu.cn (L.Z.); 2 Chongqing Engineering Research Center of Medical Electronics Technology, Chongqing 400044, China; 3 Chongqing Key Laboratory of Artificial Intelligence and Service Robot Control Technology, Chongqing 400044, China; 4 Collaborative Innovation Center for Brain Science, Chongqing University, Chongqing 400044, China; 5 Key Lab of Human-Machine Intelligence-Synergy Systems, Shenzhen Institutes of Advanced Technology, Chinese Academy of Sciences, Shenzhen 518055, China; gl.li@siat.ac.cn

**Keywords:** gesture recognition, surface electromyography, sEMG decomposition, muscle synergy

## Abstract

Currently, surface electromyography (sEMG) features of the forearm multi-tendon muscles are widely used in gesture recognition, however, there are few investigations on the inherent physiological mechanism of muscle synergies. We aimed to study whether the muscle synergies could be used for gesture recognition. Five healthy participants executed five gestures of daily life (pinch, fist, open hand, grip, and extension) and the sEMG activity was acquired from six forearm muscles. A non-negative matrix factorization (NMF) algorithm was employed to decompose the pre-treated six-channel sEMG data to obtain the muscle synergy matrixes, in which the weights of each muscle channel determined the feature set for hand gesture classification. The results showed that the synergistic features of forearm muscles could be successfully clustered in the feature space, which enabled hand gestures to be recognized with high efficiency. By augmenting the number of participants, the mean recognition rate remained at more than 96% and reflected high robustness. We showed that muscle synergies can be well applied to gesture recognition.

## 1. Introduction

Hand motion analysis is one of the most essential topics in rehabilitation for understanding and restoring human motor function, as the hand is very frequently used in our daily lives [1]. Generally, hand finger movements are controlled by the skeletal muscle of the forearms. Surface electromyography (sEMG) signals from multi-tendon forearm muscles can reflect the finger movement pattern [2,3], which is useful to finger motion classification applications such as sign language recognition [4,5] or an electromyography (EMG)-driven robotic hand exoskeleton [6]. The difference in the muscle contraction pattern that controls finger movements will alter the sEMG characteristic parameters in the time- or frequency- domain. Therefore, the recognition of hand gestures is principally based on the myoelectric feature vectors, using characteristic parameters extracted from the corresponding sEMG signals [7]. However, the robustness of this recognition is difficult to be maintained because these parameters are often affected by factors such as muscle fatigue, electrode shift, etc. [8,9,10]. Synergistic muscular activity is generally a neural-controlled strategy with high robustness for limb movement and muscle synergy patterns were successfully used for complex movement evaluations [11]. Therefore, whether the muscle synergy patterns could be broadened to hand gesture recognition left an interesting topic.

The feature set extracted from the time- and frequency-domains has received widespread interest as a powerful tool for hand movement recognition applications [12,13]. According to recent studies, better efficiency of classifications is usually achieved through a combination of multiple feature sets. Khezri and Jahed [14] proposed using two different sets of EMG features to achieve a high degree of accuracy in recognizing six distinct hand movements; one set included the mean absolute value (MAV), slope sign changes (SSC), and autoregressive (AR) model coefficients as time features, while the other set used wavelet coefficients for time-frequency representation. To increase the motion classification performance, Geng et al. [15] selected two time-domain feature sets instead, including: (1) The mean absolute value (MAV), the number of zero crossings (ZC), the number of slope sign changes (SSC), and the waveform length (WL); and (2) The six order AR model coefficients and the root mean square (RMS) amplitude of EMG signals. However, the robustness of the classification can easily be affected according to the variety of feature set combinations. The complexity of the physiological process among tasks, muscles, and participants made it difficult to quantitatively constitute stable time- and frequency- feature parameters for finger motion characterization, which was essential to maintain a high recognition rate. Huebner et al. [16] found that the location of the recording electrodes in relation to the innervation zones also affects features of the EMG signal, especially the frequency characteristics. In addition, since the sEMG signals are non-stationary and non-linear random signals [17], the robustness of the feature values also deteriorated due to individual differences, which were one of the main obstacles for the time- or frequency- domain feature analysis [18].

The hypothesis related to movements assumes that the central nervous system (CNS) generally controls muscles under an optimal activation pattern to minimize the burden on the human body induced by the behavior of the musculoskeletal system [19]. The CNS can recruit the co-activation of muscles, known as muscle synergies, to simplify the motor behavior control [20]. The existence of muscle synergies has been thought of as a common mechanism for movement control in humans, as well as in various animals such as monkeys [21] and cats [22]. The muscle synergies recruited by different neuronal pathways have been implemented in biomedical applications, such as motor behavioral evaluations [11,23] and movement control for disabled patients [24,25]. For example, Jiang et al. [26] proposed an algorithm extracting neural control information from sEMG based on the synergy theorem, to drive myoelectric prostheses performing upper limb movements in multiple degrees of freedom [27]. Sharon et al. [28] incorporated a modified non-negative matrix factorization (NMF) algorithm to analyze the synergies from the sEMG signals of aged participants, which established the representative set of synergies for controlling movements in multiple directions. Moreover, as a direct approach of extracting information based on the physiological neural-controlled strategy, muscle synergy analysis can provide low dimensional control signals with robustness. Lunardini et al. [29] demonstrated in an upper limb movement study with a myoelectric control system that the synergy-based approach exhibited higher robustness than the traditional muscle-pair approach for controlling. Ma et al. [25] also agreed that the control scheme was more robust for multi-grasp movements based on synergy analysis, which facilitated prostheses making a better performance.

Recently, the characteristic analysis of finger movements has attracted increasing interest for its high potential in rehabilitation. For hemiplegic patients with a unilateral motor disability, hand motion from the healthy side could be implemented to drive the exoskeletal rehabilitation system to assist training finger movements in the affected side. During this process, the analysis of the detection and recognition of finger motion plays a key role. Pioneering studies [30] have involved several techniques, for example, Raheja et al. [31] proposed controlling robots using hand gestures captured by a live camera with an imaging processing method; meanwhile, Li et al. collected sEMG signals and designed a prosthetic hand with Principal Component Analysis and Deep Learning methods [32]. However, there is little research considering hand gesture recognition with the inherent physiological mechanism of muscle synergies. This study extracted the synergistic patterns of forearm multi-tendon muscles for gesture classification. Five hand gestures most frequently used in daily activities [33] were selected and the sEMG from six muscles corresponding to the assigned postural performances were recorded. A non-negative matrix factorization (NMF) algorithm was used to obtain the myoelectric synergy pattern and the support vector machine (SVM) was employed to investigate the feasibility of muscle synergy in the recognition of different finger motions.

## 2. Methods

### 2.1. Participants

Five healthy volunteers (mean age: 24.2 ± 1.2 years; height: 175.1 ± 9.8 cm; weight: 65.62 ± 8.1 kg) were enrolled in the study. All participants were right hand dominant. No evidence of skeletal, muscle, or neurological diseases were reported before participation commenced. The protocol of this study was approved by the Institutional Review Board of Shenzhen Institutes of Advanced Technology, Chinese Academy of Sciences. All participants signed an informed consent form before the experiment.

### 2.2. Experimental Protocol

**Figure 1:**
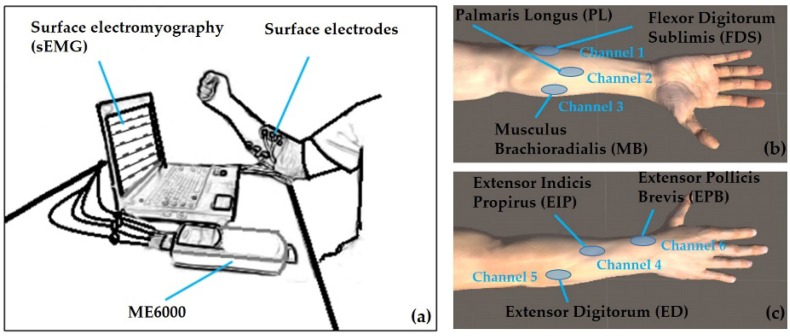
The experiment set-up for the sEMG recording during the hand movement. (**a**) A diagram of the experiment scene. The surface electrodes placement were configurated to muscles as Channel (Muscle): (**b**) Channel 1 (FDS), Channel 2 (PL), Channel 3 (MB) and (**c**) Channel 4 (EIP), Channel 5 (ED), Channel 6 (EPB).

**Figure 2:**
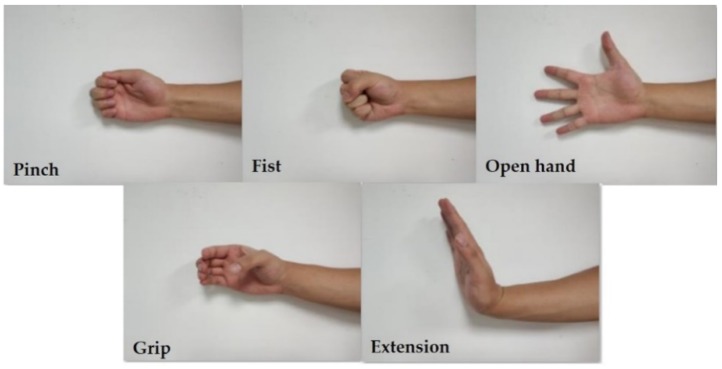
The hand gestures involved for the task performances: Pinch (P), Fist (F), Open hand (O), Grip (G), and Extension (E).

Similar to our previous work [12], the participant sat naturally in a chair with their right elbow joint kept on the table and their forearm flexed to a 90-degree angle, as shown in Figure 1. Five of the most frequently used gestures (Figure 2) in daily life [34,35,36] were designed for the task performance, including: pinch (P), fist (F), open hand (O), grip (G), and extension (E). The experiments utilized a repetitive block design. The participants performed the tasks following the visual and verbal instructions given by the experimenter. For every trial, the specific finger movement was performed and held for 4 s, followed by a 5 s relaxation period to avoid fatigue, and each block was repeated 40 times per gesture task.

### 2.3. Electromyography

The EMG activity was acquired from six forearm muscles using the surface EMG system (ME6000, Mega Electronics Ltd, Kuopio, Finland). The muscles involved in this study included (Figure 1b,c): (1) Flexor digitorum sublimis (FDS), (2) palmaris longus (PL), (3) musculus brachioradialis (MB), (4) extensor indicis propirus (EIP), (5) extensor digitorum (ED), and (6) extensor pollicis brevis (EPB). The skin was cleaned with alcohol and shaved before the experiment [11,36]. An elastic gauze was used to fix the patch electrode to the skin surface steadily. The recording system bandwidth was set to 15–500 Hz during signal collection and the sampling rate was 1 kHz.

### 2.4. Data Analysis

#### 2.4.1. EMG Preprocessing

Offline data analysis was performed using Matlab 2014a (The MathWorks, Inc., Natick, MA, USA). The noise and power frequency interference were removed from the collected sEMG data with a 20–500 Hz band-pass filter and a 50 Hz notch filter. The envelope was extracted through a 3 Hz low-pass filter. The activity intensity of the filtered six-channel myoelectric envelope signal was calculated to detect the onset of movements as following [37]:

(1) $$EMG_{average}=\sqrt{\sum_{i=1}^{6}EM{G_{\left(i\right)}}^{2}}$$

(2) $$EMG_{average}\{\begin{array}{cc} \geq 0.005 & active\\ else & rest \end{array}$$

where $EMG_{\left(i\right)}$ is the envelope signal corresponding to the *i*th channel (*i* = 1, 2, 3, 4, 5, 6) and $EMG_{\left(average\right)}$ is the squared mean value of the envelope signals of the six channels, with a threshold of 0.005. The myoelectric activity was determined to start when the $EMG_{\left(average\right)}$ was above the threshold (referred to as the onset of movement) and the following 4 s segment was selected as the valid sEMG of one task trial. The EMG envelope signal for gesture performance was extracted from the 4 s sEMG data, trial by trial. Data down-sampling was then performed and the sample set was constituted for subsequent EMG pattern recognition.

#### 2.4.2. Non-Negative Matrix Factorization (NMF) Algorithm

The NMF algorithm [38] was used to extract muscle synergies and the corresponding activation weights by decomposing the resultant sEMG envelope as:

(3) V=W×H

where *V* is the m×n envelope signal matrix of each trial (*n* is the length of the muscle activation pattern, m is the number of muscles (six muscles)), *W* is a m×s matrix (s is the number of muscle synergies, 1 ≤ *s* ≤ 6) indicating the synergy pattern between the six muscle channels, and *H* is the s×n coefficient matrix representing the modulation and contribution of specific muscle synergy.

**Figure 3:**
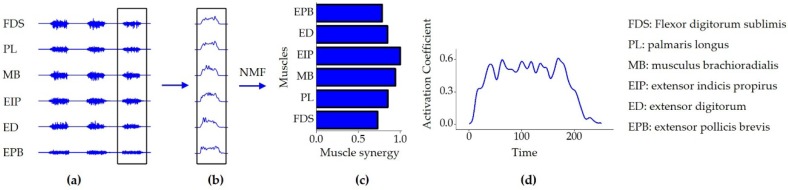
An example of the Non-Negative Matrix Factorization (NMF) decomposition to electromyography (EMG) signals. (**a**) The original surface electromyography (sEMG) signals acquired from six muscle channels of one participant when performing gesture F, (**b**) the extracted envelope EMG signal, (**c**) the muscle synergy when s = 1, and (**d**) the corresponding activation coefficient curve (H) when s = 1. The channel-muscle configurations were illustrated in Figure 1 as (1) FDS, (2) PL, (3) MB, (4) EIP, (5) ED, and (6) EPB.

Therefore, each column of *W* represents the weights of the corresponding muscle for a single synergy and each row of *H* represents the extent to which the corresponding synergy is activated or generated. An example of the EMG signal and its NMF decomposition is illustrated in Figure 3.

The number of muscle synergies (s) was optimized by calculating the Variability Accounted For (VAF) between the envelope signal data matrix (*V*) and the reconstruction matrix ($V^{\prime }=W\times H$) [39], following the equation as below:

(4) $$\mathrm{VAF}=1-\frac{{\left(V-V^{\prime }\right)}^{2}}{V^{2}}$$

The number of muscle synergies should be selected appropriately in order to keep the original information as much as possible. The optimal number of muscle synergies was set as the minimum number of synergies that satisfied the criteria of (1) the mean global value of the VAF > 95%, and (2) the mean global VAF increase <1% when adding another synergy [40].

#### 2.4.3. EMG Feature Vector Construction and Classification

After the muscle synergy matrix was extracted via the NMF algorithm, the feature vector was structured with the weights of each muscle in a synergistic matrix as follows:

(5) $$A=\left[A_{1},A_{2},\cdots ,A_{6}\right]$$

where $A_{i}$ is the feature of the *i*th channel (i=1,2,⋯,6), which represented the weights of the *i*th muscle channel while a gesture task was conducted. In other words, each hand gesture is characterized by a synergy feature vector of size 6 × 1.

The support vector machine (SVM) method was widely employed for the gesture recognition [41,42,43]. After the feature vectors were extracted for each gesture movement, the extracted feature set was divided into a training set and a test set. The SVM model was trained and stored in CSVMStruct, which saved various parameters of the classifier. The model was applied to the gesture classification using a test set with the 5-fold cross validation (CV) method.

#### 2.4.4. The Distance of Different Gesture Feature Sets

The feature vector can be represented through a point in the feature space, therefore, we used Equation (6) to find the centroid of every gesture feature set. Then, due to the distance of Equations (7) and (8), we calculated the distance between the centroids of one gesture to each distributed point of another gesture (Equation (7)), and the distance between different gesture centroids (Equation (8)) in the feature space.

(6) $$A_{c}=\frac{1}{N}\overset{6}{\underset{i=1}{\sum }}A\left(i,:\right)$$

(7) $$D_{B-A_{c}}\left(j\right)=\sqrt{{\left[B\left(j\right)-A_{c}\right]}^{2}},\;j=1,\;2,\;\ldots \ldots ,\;N$$

(8) $$D_{B_{c}-A_{c}}=\sqrt{{\left[B_{c}-A_{c}\right]}^{2}}$$

where *i* represents the muscle channels (i=1,2,⋯,6) and *N* is the sample size of the gestures (*N* = 40). *A* and *B* represent the data samples obtained from gestures *A* and *B*, respectively. Subscript *c* represents the centroid, with $A_{c}$ being the centroid of gesture *A* and $B_{c}$ being the centroid of gesture *B*. *D* represents the distance in the feature space, as $D_{B-A_{C}}\left(j\right)$ is the distance from the *j*th data sample of the *B* class gesture to the $A_{c}$ and $D_{B_{c}-A_{c}}$ is the distance between the centroids of gestures *B* and *A*.

## 3. Results

### 3.1. Selection of the Optimal Number of Muscle Synergies

**Figure 4:**
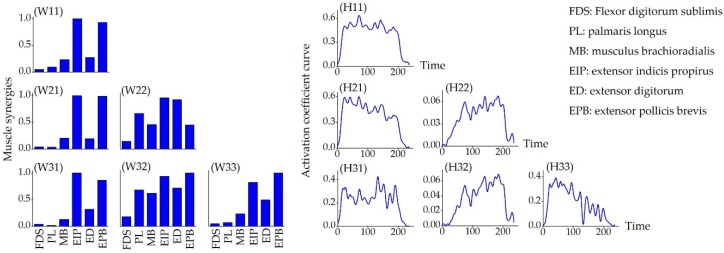
The muscle synergy patterns (left) and the activation coefficient curves (right) for one gesture (gesture E) as the number of synergies increased (*s* = 1 (first row), 2 (second row), 3 (third row)). The channel-muscle configurations were illustrated in Figure 1 as (1) FDS, (2) PL, (3) MB, (4) EIP, (5) ED, and (6) EPB. The NMF algorithm was used to extracted *W* and *H* matrix according to equation (3), where *W*ij was the *j*th muscle synergy matrix when number of synergies equal to *i* and *H*ij was the corresponding coefficient matrix representing contribution of the specific muscle synergy as well (I=s=1,2,…,6, and j=1,2,…,i).

Figure 4 shows the muscle synergy patterns, as the number of synergies (*s*) increased from one to three, during hand extension (gesture E). New synergy patterns were observed when the synergy number increased and the corresponding activation coefficient curve was altered as well. However, the 1st muscle synergy mode was relatively robust to the variations introduced by muscle synergy changes (an example is illustrated by W11 (*s* = 1), W21 (*s* = 2), and W31 (*s* = 3) in Figure 4).

**Figure 5:**
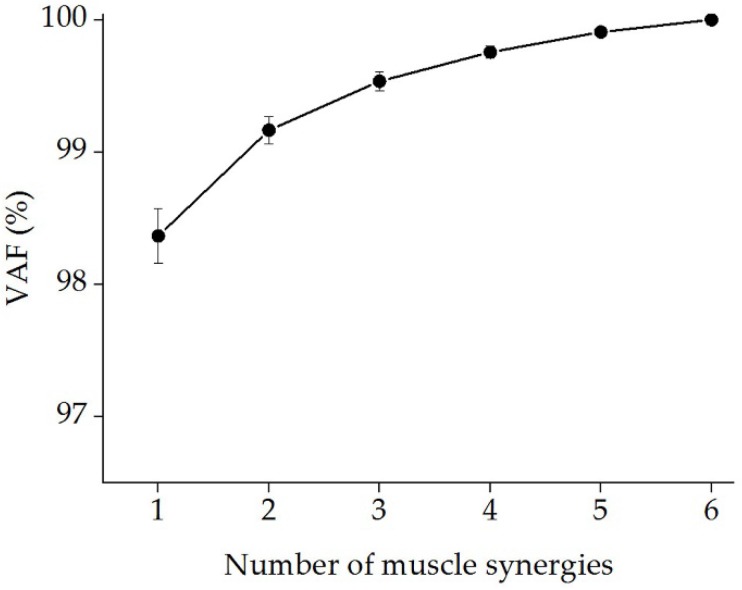
The relationship between the mean Variability Accounted For (VAF (%)) and the number of muscle synergies. The mean global VAFs were calculated using pooled data from all participants. According to criteria defined in Section 2.4.2, the optimal number of synergies was determined as *s* = 1.

**Figure 6:**
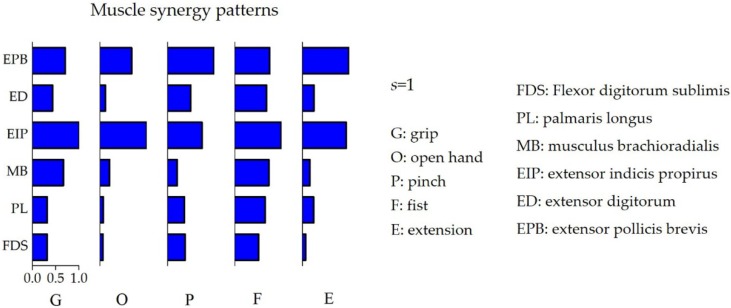
The muscle synergy patterns according to the gestures grip (G), open hand (O), pinch (P), fist (F), and extension (E). The optimal number of synergies was set as *s* = 1 for all five of the gestures. The blue colored bars indicate the synergy matrix (*W*), which shows the extent of contribution changes between the six muscles when performing the corresponding gesture.

Figure 5 shows the mean VAF as a function of the number of muscle synergies. The mean values of the VAF of the five gestures were 0.984 ± 0.4 when s = 1 and 0.992 ± 0.2 when s = 2. According to the criteria mentioned in Section 2.4.2, s = 1 was selected as the optimal synergy number. As illustrated in Figure 6, we extracted the muscle synergy patterns gesture by gesture. Hand gestures with different finger motions exhibited different muscle activation patterns among the forearm multi-tendon muscles. For example, EIP and EPB provided the primary contribution during hand opening (gesture O), while the other four muscles contracted at a lower level. The hand grip (gesture G) mainly activated MB, EIP, and EPB, while the muscles of EIP, ED, and EPB dominated the motor control for the pinch gesture (gesture P). In addition, all six muscles were activated simultaneously to a high extent to compose the corresponding synergy patterns for the fist gesture (gesture F).

### 3.2. Clustering Effect of the Feature in Feature Space

**Figure 7:**
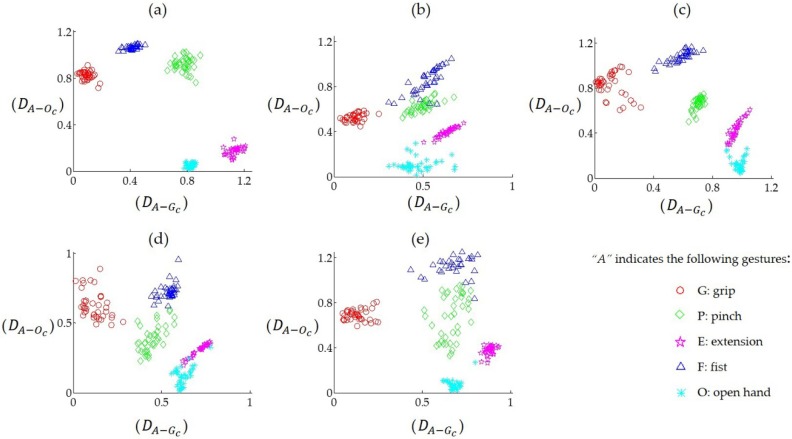
The distribution of the gesture features illustrated by the distances from the data points to the specific centroids (defined by Equation (7)) in the feature space. The x-axis ($D_{A-G_{c}}$) was the distance from the data samples of the A class gesture to the centroid of the grip gesture (G) and the y-axis ($D_{A-O_{c}}$) was the corresponding distance between data samples of A gesture to the centroid of the open hand gesture (O). Sub-plots of (**a**–**e**) present one participant each. Different gestures can be visually distinguished from the scatter plots.

**Figure 8:**
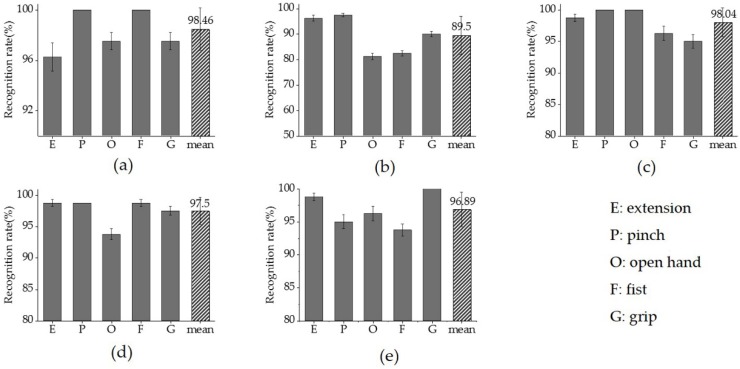
The gesture recognition rate results, with (**a**–**e**) representing one individual participant each. The extracted feature data was divided into a training set and a test set at the ratio of 4:1 and the recognition rate of each gesture (solid) was calculated through a support vector machine (SVM) classifier with 5-fold cross-validation. The mean recognition rates (shadow) for every participant were also calculated.

Figure 7 shows the distribution of synergy-based gesture features in the feature space. As illustrated in Figure 7, the five gestures have been clustered and can be visually distinguished from each other. Figure 8 shows the recognition results for different gestures of individual participants, where the mean recognition rate ranged from 89.5 ± 7.5% to 98.5 ± 1.7%. Gestures G, E, and P generally had higher recognition rates (>95%) than gestures O and G. However, the recognition rates of different gestures across individuals varied, as gesture O in Figure 8d and gesture F in Figure 8e represent lower rates than other gestures in these participants (<95%), while the lowest rates were observed according to gestures O and F for the second participant (<90%, Figure 8b).

### 3.3. Classification Performance of Features

**Figure 9:**
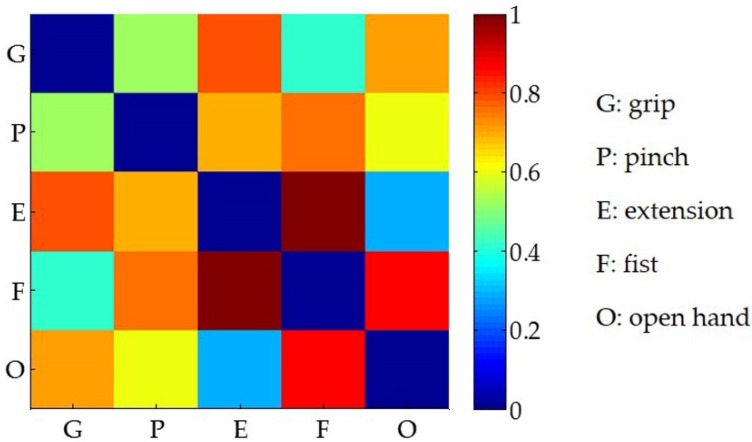
The distance between the centroids (Equation (8)) across the five gestures of all participants. All of the distance values were normalized.

**Figure 10:**
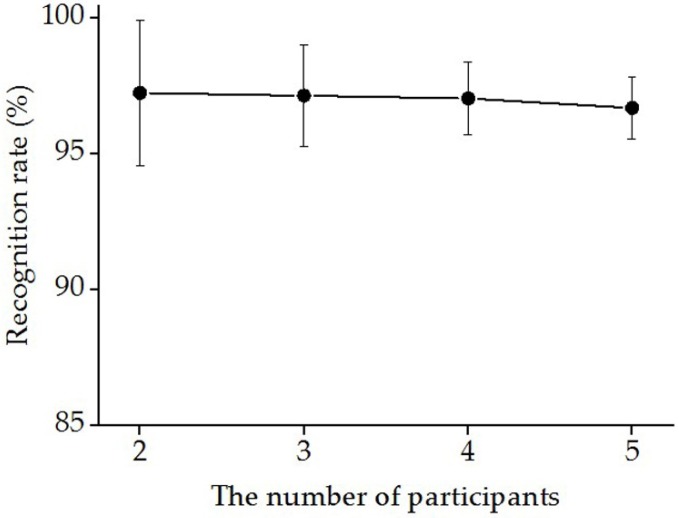
The relationship between the recognition rate and the participant population. The extracted feature data were pooled and divided into a corresponding training set and test set. The recognition rate was calculated through a support vector machine (SVM) classifier with 5-fold cross-validation. All combinations of the participants were considered.

Figure 9 shows the normalized distance of the centroid (Equation (8)) across the five gestures when pooling all participants as a whole. The results indicated that the synergistic feature clusters differed from each other according to the distances in the feature space. In order to explore the impact of the sample size on gesture classification, we calculated the gesture recognition rate by augmenting the number of participants from two to five. As shown in Figure 10, the change in the number of participants had no significant effect on the mean recognition rate, but the corresponding deviation decreased as the number of participants increased. Meanwhile, the mean recognition rate remained at about 96% without significant fluctuations during the augmentation of participants.

## 4. Discussion

The presented study investigated the feasibility and efficiency of hand gesture recognition using a feature set based on muscle synergy pattern analysis. A Non-Negative Matrix Factorization (NMF) algorithm was applied to extract the myoelectric synergies of forearm muscles, and the muscle synergy feature vectors with low dimensionality were constructed for hand gesture recognition. Our preliminary results revealed that each of the five gestures exhibited stable muscle synergies and different hand gestures could be characterized by a certain co-activation pattern of forearm muscles. Furthermore, with support vector machine (SVM) classification, a high recognition rate (>96%) can be achieved for the five gestures, which suggests that the synergy-based constituted feature vector matrix has the potential to be incorporated for better recognition of finger motions.

Our study found that hand postures could be characterized by a specific muscle synergy pattern, which demonstrated a stable relationship between finger movement and the co-contraction of multi-tendon forearm muscles. Previous works of multi-finger quick force production confirmed that co-activation of muscle, as the synergy pattern that multiple functional muscle units recruited by the central nervous system [44], is a fundamental neural strategy for motor control [45]. Research also suggested that muscle synergies can give insight into the complex coordination of multiple muscles [19]. Therefore, the feasibility of forearm muscle synergies being implemented for controlling finger movement in multiple degrees of freedom attracted interest. D’Avella et al. [46] established that the combination of a few time-varying muscle synergies can describe muscle patterns for reaching movement, which suggested that the muscle synergies were the basis of arm movement control function under a modular architecture.

All of the hand gestures tested in this study were dominated by co-activation of six forearm muscles (FDS, PL, MB, EIP, ED, and EPB). Our results showed that multi-tendon muscle played different roles due to variations in the specific finger gesture task requirements. An average contribution of 0.72 could be achieved for all six muscles for gesture F (Figure 6), suggesting that the motor control required participation of more forearm muscles to conduct a fist. Meanwhile, for gesture O, EIP and EPB made the primary contributions (0.95 and 0.99, respectively), which was in accordance with the inherent physiological mechanism that the contribution of finger motor control varies with the task performance [47]. In addition, the ED muscle, located in the posterior forearm, primarily controls stretching movements of the finger, wrist or elbow [48], which theoretically supports our results for the hand open gesture, as presented in Figure 6.

With synergy-based features from multi-tendon forearm muscles, the performance of hand motion classification reached high accuracy and robustness. As Figure 8 addressed, the gesture recognition rate results for individual participants shows that each gesture had a good recognition effect. Similar results were found with distance analysis, which was represented as visually distinguishable clusters in the feature space (Figure 7) and the distance of the centroid across the five gestures of all participants (Figure 9). In regards to the distance clusters in Figure 7, the five different gestures can be partitioned to each other in the feature space, though centroid of each cluster shifts due to individual differences, however, the relative positions were maintained well. This is perhaps in accordance with the physiological mechanism that the performance of different movements generally co-activated differently, while the same behaviors corresponded to specific muscle synergy patterns, which was important for the robustness of the motion identification. Specifically, the distribution of the gesture features of participant (a) fragmented into much smaller clusters, with an obviously lower inter-cluster variation compared to the rest of the participants. The reason behind these clustering improvements was probably due to participant (a) training much more frequently than the others, which inspired promising further improvements via the exercise training. As Figure 10 addressed, the gesture recognition rate was over 96%, and no significant fluctuations were observed when the number of participants was increased from two to five. This shows that the accuracy of the recognition rate maintained a stable performance, despite an increase in the sample complexity. Our results also implied that, based on muscle synergy, the robustness can be maintained even with significantly reduced training samples. Therefore, the feature set based on the synergies presented its advantage in simplifying the hand motion classification with appropriate accuracy and robustness, using minimal training sets and calculation.

The feature vectors were constructed with low dimensionality, in response to the neural control strategy that the muscle synergies implemented. Usually, feature analysis in the time-domain or frequency-domain involves feature sets with high dimensionality to achieve high accuracy in hand motion classifications [49]. To reduce the dimensionality of the feature vector, Khushaba et al. [50] proposed an optimized feature projection technique, combining the root mean square (RMS) and autoregressive coefficients. However, fine motor behaviors are controlled under specific muscle synergy patterns [51], and the synergy can facilitate motor control in a simplified way, which reduced the dimension of the corresponding synergy-constituted feature vectors. Therefore, with consideration of the physiological mechanism of movements, the synergy-based feature set minimized the dimensionality of the feature vector to improve the hand movement classification.

In conclusion, we performed a pioneering study, employing muscle synergy analysis which was based on a neural-controlled strategy to optimize hand gesture recognition. With an NMF algorithm, the synergistic activation pattern of forearm multi-tendon muscles has been extracted for hand gestures. The muscle synergy pattern can be used to construct the feature vector for gesture classification with high efficiency. The present study suggested that, by means of the mechanism of muscle synergy, hand gesture recognition can be achieved with lower feature vector dimensionality and high robustness. However, only a few hand motion tasks using limited participants have been tested here, and further work will extend the investigation, with an enlarged population and a broadened variety of hand motor skills using a combination of finger movement and wrist rotation.
